# Non-Destructive Quantitative Analysis of Grape Components Based on Visible–Near-Infrared/Shortwave–Near-Infrared Spectroscopy and Chemometrics

**DOI:** 10.3390/foods15213854

**Published:** 2026-10-28

**Authors:** Kai Wu, Chengli Hao, Wei Guo, Zhiwei Li, Decong Zheng

**Affiliations:** 1 College of Agricultural Engineering, Shanxi Agricultural University, Jinzhong 030801, China; wukai@sxau.edu.cn (K.W.); guowei@sxau.edu.cn (W.G.); 2 Dryland Farm Machinery Key Technology and Equipment Key Laboratory of Shanxi Province, Jinzhong 030801, China; haochengli@sxau.edu.cn; 3 College of Food Science and Engineering, Shanxi Agricultural University, Jinzhong 030801, China; 4 College of Information Science and Engineering, Shanxi Agricultural University, Jinzhong 030801, China

**Keywords:** grape, VNIR/SWIR spectroscopy, chemometrics, feature extraction, non-destructive quantitative analysis

## Abstract

The contents of sugar and acid components in grapes are important indicators for evaluating their quality and maturity. To achieve rapid, non-destructive, and accurate detection of glucose, fructose, tartaric acid, and malic acid in grapes, this study selected 60 mature grape varieties as research objects. Spectral data were acquired using a visible–near-infrared/shortwave–near-infrared (VNIR/SWIR) hyperspectral system, and the contents of the four components were determined by high-performance liquid chromatography (HPLC) as reference values for modeling. Based on spectral preprocessing and CARS-based feature-variable selection, PLSR, SVM, and BPNN prediction models were constructed using full variables and selected feature variables, respectively, and their performance was evaluated using R^2^, RMSE, and RPD. The results showed that the feature-variable models outperformed the full-variable models, with the NIR/SWIR-CARS-SVM model performing best, with R_P_^2^ values reaching 0.8630, 0.8441, 0.8546, and 0.8283; RPD_P_ values reaching 2.7287, 2.5782, 2.6451, and 2.4145; and RMSE_P_ values as low as 3.5114, 3.4751, 0.5310, and 0.1646 for glucose, fructose, tartaric acid, and malic acid, respectively. This study provides an effective method for the non-destructive quantitative detection of sugar and acid components in grapes. Under the single-site, single-season conditions of this trial, the 60 varieties differed substantially in glucose, fructose, tartaric acid, and malic acid. These findings suggest variety-level differences that warrant further validation across multiple vintages and geographical regions before industrial recommendations can be made.

## 1. Introduction

The grape (*Vitis vinifera* L.) is a commercially significant fruit species that can be consumed fresh or processed into juice, raisins, and wine [1]. Grape berries accumulate substantial amounts of soluble sugars, organic acids, and minerals, contributing to their notable nutritional value [2]. Harvest quality of table grapes centers on high sugar, low acidity, and rich aroma, while sugar and acid contents in processing grapes significantly influence finished product flavor [3]. Therefore, quantitative analysis of sugars and acids at fruit maturity is crucial for determining the utilization value, quality grade, and economic value of the fruit.

Total soluble sugars and total acids are commonly used as indices for evaluating grape quality and maturity. However, grapes with identical total sugar and total acid contents may nonetheless exhibit distinct flavor profiles. Specifically, studies have shown that glucose and fructose are the main soluble sugars in grapes, together accounting for approximately 98.9% of total sugars, while tartaric acid and malic acid are the main organic acids, together accounting for more than 84.4% of total acids [4]. Differences in the relative proportions of these major sugars and organic acids can lead to differences in sensory properties. For example, fructose is approximately twice as sweet as glucose; therefore, at the same total sugar content, grapes with a higher fructose proportion taste sweeter. Similarly, tartaric acid has a sharper sourness, whereas malic acid has a milder sourness; thus, at the same total acid content, differences in acid composition can also lead to differences in sensory perception. For producers, total sugar and total acid contents alone are insufficient to determine which specific sugar or acid component should be targeted for regulation; for consumers, differences in sugar and organic acid composition directly affect flavor perception and acceptance. Therefore, determining glucose, fructose, tartaric acid, and malic acid is essential for capturing the compositional basis of grape flavor and for guiding targeted regulation of sugar and acid composition beyond total sugar and total acid contents.

Conventional assessment of grape sugar and acid levels has largely depended on wet-chemical methods. Fehling titration and indicator-based titration, for example, are cost-effective but provide only total sugar and total acidity values rather than component-specific quantification [5,6]. Enzymatic methods can separate glucose from fructose, yet they need two separate assays [7]. High-performance liquid chromatography (HPLC) can simultaneously quantify various sugars and acids with high precision, but it requires complex steps such as sample preparation, chromatography, and data analysis, and is expensive and operator-intensive [8]. Additionally, all these techniques are destructive and time-consuming [9]. Therefore, a non-destructive, cost-effective, and convenient detection method is urgently needed to achieve accurate determination of sugar and acid components in grapes.

Against the backdrop of rapid agricultural informatization, spectroscopic analysis has been applied in the quality of agricultural products, owing to its convenience, efficiency, and non-destructive nature [10]. Spectroscopic analysis is based on the absorption, emission, scattering, or transmission characteristics of matter with respect to the electromagnetic radiation of different wavelengths, enabling the acquisition of information on its composition, structure, and concentration [11]. Numerous studies have confirmed the utility of this approach. Xia et al. [12], in their predictive study on the soluble solids content of apples, demonstrated that the combination of visible–near-infrared spectral data with optimized algorithms enables rapid, accurate, and non-destructive determination of soluble solids content, thereby providing support for apple quality evaluation. Fang et al. [13] showed that combining spectroscopy with machine learning allows for rapid, non-destructive, and simultaneous multi-component detection of juice adulteration, though it still suffers from matrix interference and limited model generalizability. Zhou et al. [14] employed visible–near-infrared spectroscopy combined with machine learning and deep learning algorithms to predict key quality indicators of grapes, including soluble solids content (SSC), total acidity (TA), and total phenols (TPs). They achieved favorable results, confirming that this method can be used for the rapid and accurate assessment of grape maturity. He et al. [5] also employed visible–near-infrared spectroscopy combined with machine learning algorithms to perform predictive modeling of SSC, TA, and pH value in grapes, providing an effective approach for rapid detection.

The aforementioned studies have confirmed the potential of spectroscopic techniques for non-destructive information acquisition in agricultural products. However, for grape berries, existing research has focused only on total sugar and total acid levels, without achieving the differentiation and quantification of individual monosaccharides and organic acids. Based on this, the present study proposes to develop a rapid, non-destructive, and cost-effective spectroscopic detection strategy for the simultaneous and accurate quantification of glucose, fructose, tartaric acid, and malic acid in grapes.

The specific research procedures to be adopted are as follows: First, grape samples were collected and properly stored. Subsequently, spectral data were acquired using a VNIR/SWIR hyperspectral system, and raw spectral data were recorded. The contents of glucose, fructose, tartaric acid, and malic acid were then determined by HPLC as reference values for modeling. Next, multiple spectral preprocessing methods were applied to the raw spectra, the optimal one was determined, and feature variables were selected using the CARS algorithm. Finally, PLSR, SVM, and BPNN prediction models were constructed based on full variables and selected feature variables, respectively, and their performance was compared using R^2^, RMSE, and RPD as evaluation metrics to identify the optimal model and validate its generalization ability. Through the above steps, this study aims to provide an effective method for the rapid, non-destructive, and accurate detection of glucose, fructose, tartaric acid, and malic acid in grapes, and to offer technical support for grape quality evaluation and quality control.

## 2. Materials and Methods

### 2.1. Experimental Materials

The experimental materials used in this study were obtained from the Taigu Grape Nursery of the National Fruit Tree Germplasm Repository, located at the Institute of Pomology, Shanxi Agricultural University (112°48′ E, 37°38′ N), which is currently the germplasm repository with the most abundant collection of grape varieties in China. A total of 60 grape varieties were collected for this experiment, covering both table grape and wine grape cultivars, with skin colors ranging from red, pink, purplish red, yellowish green, and blue-black, to ensure the representativeness of the samples.

During harvest, whole grape clusters were cut from the vine with pedicels retained to minimize moisture loss and were immediately stored under refrigeration. Prior to data acquisition, 30 intact and size-uniform berries were selected, of which 15 were divided into three groups for spectral acquisition, and the remaining 15 were used for the determination of glucose, fructose, tartaric acid, and malic acid contents. A total of 180 sets of averaged spectral data and 60 sets of chemical compositional data were obtained.

### 2.2. Hyperspectral Images Acquisition and Extraction

#### 2.2.1. Hyperspectral Image Acquisition

Hyperspectral images were acquired using a Headwall Photonics Starter Kit system (USA), which comprises a halogen illumination source, a dark enclosure, a motorized platform, and dual lenses.

Lens 1 covers 380–1000 nm with a spectral resolution of 0.727 nm across 856 bands, whereas Lens 2 spans 900–1700 nm with a resolution of 4.715 nm over 172 bands. Marginal bands were excluded to avoid errors arising from unstable reflectance at the spectral edges. For subsequent modeling, the effective range of Lens 1 was set to 430–900 nm (646 bands), encompassing the Visible Light (VIS, 430–700 nm) and Near-Infrared (NIR, 700–900 nm) regions, while Lens 2 was restricted to 950–1650 nm (148 bands), all within the Short-Wave Infrared (SWIR) spectrum.

Prior to spectral acquisition, the instrument is warmed up and calibrated. Subsequently, grape berries are placed on the sample stage, and spectral data are collected through Lens 1 and Lens 2, respectively. After completing measurements for one variety, the instrument is recalibrated to ensure data quality.

Previous studies have indicated that the prediction model built by directly concatenating spectral data from Lenses 1 and 2 outperformed models based on a single lens. Furthermore, extracting feature variables from each lens separately using the CARS algorithm and then fusing them yielded better model performance than using all variables. Therefore, this study adopted this strategy to achieve the accurate quantification of grape components [15,16,17].

#### 2.2.2. Spectral Data Extraction

To simplify the extraction process of hyperspectral image data and enable batch processing, this study developed a sampling point selection software using Visual Basic (version 6.0, Microsoft Corporation, Redmond, WA, USA) to select regions of interest (ROIs) [18]. Subsequently, the generated sampling point files were imported into Spectral View software 5.5.1(Headwall Photonics, Bolton, MA, USA) to extract spectral data from the corresponding regions. Based on the appearance of the grape berries, the ROI was defined as an elliptical shape, covering more than 18,000 pixels. Specifically, the formula for calculating the elliptical ROI mask is as Equation (1).

(1) $$\frac{{\left(x-x_{0}\right)}^{2}}{a^{2}}+\frac{{\left(y-y_{0}\right)}^{2}}{b^{2}}\leq 1$$

where *x* and *y* are the pixel coordinates in the image, (*x*_0_, *y*_0_) is the center of the ellipse, and *a* and *b* are the semi-major and semi-minor axes of the ellipse, respectively.

Reflectance of grape samples was obtained by calculation the average reflectance for all pixels, the calculation formula is as Equation (2).

(2) $$T=\frac{1}{n}\sum_{i=1}^{n}T_{i}$$

where *T* represents the average reflectance, *T_i_* represents the reflectance of the *i*-th pixel point, and *n* denotes the number of pixels. The calculated average reflectance will serve as the foundational dataset for subsequent data processing.

### 2.3. Laboratory Measurement of the Chemical Parameters

The relevant chemical parameters were determined by high-performance liquid chromatography (HPLC) using a Waters 2695 HPLC system (Alliance^®^, Waters Corp., Milford, MA, USA).

#### 2.3.1. Extraction Procedure

The sample extraction procedure was carried out as follows:

(1) Take grape berries, remove the stems and seeds, and then homogenize the pulp and skins together.
(2) Accurately weigh 20.0 g of the grape homogenate sample into a homogenizer, add 20 mL of ultrapure water, and homogenize thoroughly until no obvious tissue fragments remain, then transfer the mixture to a centrifuge tube and vortex thoroughly to mix.
(3) Centrifuge at 4 °C and 5000 r/min for 5 min, and carefully pipette off the supernatant. This aqueous extract supernatant will be used for the subsequent purification of sugars and organic acids, respectively.
(4) Purification of sugar samples: Accurately pipette 1.0 mL of the above aqueous extract supernatant, add 1.0 mL of acetonitrile, and vortex to mix. Centrifuge at 4 °C and 8000 r/min for 5 min. Take the supernatant and pass it through a 0.45 μm organic-phase microporous filter membrane. Transfer the filtrate to a sample vial and label it as the “sugar test solution”.
(5) Purification of organic acid samples: Separately, accurately pipette 1.0 mL of the aqueous extract supernatant, add 1.0 mL of phosphate buffer (10 mmol/L KH_2_PO_4_, pH 2.4), and vortex to mix. Centrifuge at 4 °C and 8000 r/min for 5 min. Take the supernatant and pass it through a 0.45 μm aqueous-phase microporous filter membrane. Transfer the filtrate to a sample vial and label it as the “acid test solution”.

#### 2.3.2. HPLC Determination of Glucose and Fructose

Install an amino chromatographic column (XB-NH2 column, 250 × 4.6 mm, 5 μm, 40 °C) and perform detection with a refractive index detector. Parameter settings: the mobile phase is an acetonitrile–water solution (75:25, *v*/*v*), filtered through a 0.45 μm membrane and ultrasonically degassed before use; flow rate 1 mL/min; column temperature 40 °C; injection volume 20 μL; run time 30 min.

#### 2.3.3. HPLC Determination of Tartaric Acid and Malic Acid

Install an acid-resistant chromatographic column (AQ-C18 column, 250 × 4.6 mm, 5 μm, 40 °C) and perform detection with a diode array detector at a detection wavelength of 210 nm. Parameter settings: the mobile phase is a mixture of 0.02 mol/L potassium dihydrogen phosphate buffer (pH 2.50) and methanol at a volume ratio of 98:2; flow rate 1 mL/min; column temperature 40 °C; injection volume 20 μL; run time 20 min.

#### 2.3.4. Expression of Results

Glucose, fructose, tartaric acid, and malic acid were identified by comparing their retention times with those of the corresponding standards under identical chromatographic conditions. Quantification was performed using the external standard method. Under chromatographic conditions identical to those used for the samples, linear regression was performed with the concentrations of a series of standard solutions as the *x*-axis and the corresponding peak areas as the *y*-axis to construct calibration curves. The sample peak areas were then substituted into the calibration curves to calculate the contents. The formulas for calculating the calibration curve, the concentration of the target compounds in the sample injection solution, and the content of the target compounds in the original sample are as Equations (3)–(5).

(3) A=kC+b

(4) $$C_{sample}=\frac{\left(A_{sample}-b\right)}{k}$$

(5) $$Content=\frac{C_{sample}\times V\times D}{m}$$

where *A* represents the chromatographic peak area of the standard, *C* is the standard concentration (mg/mL), *k* is the response factor, *b* is the intercept, *A_sample_* is the peak area of the sample solution, and *C_sample_* is the concentration of the sample injection solution (mg/mL); and *V* is the final extract volume (mL), *D* is the dilution factor, and m is the sample mass (g). Results are expressed on a fresh weight basis and reported as mean ± standard deviation (SD) of three independent replicates (*n* = 3) [19,20,21].

By employing this method, the present study conducted sample preparation, chromatographic separation, and quantitative calculation, thereby achieving accurate quantification of glucose, fructose, tartaric acid, and malic acid.

### 2.4. Spectral Data Preprocessing and Dataset Partitioning

#### 2.4.1. Spectral Data Preprocessing

To eliminate interferences caused by environmental conditions, instrument performance, and random noise, and to enhance the correlation between reflectance data and physicochemical indicators, preprocessing of the raw spectra can effectively improve the signal-to-noise ratio, thereby ensuring the stability and accuracy of the model. In this study, the spectral data were preprocessed sequentially using three methods: Savitzky–Golay convolutional smoothing (S-G), standard normal variate (SNV), and multiplicative scatter correction (MSC). The S-G method, based on local polynomial fitting, aims to smooth the spectral curves, remove random noise, and enhance signal continuity and stability [21]. The SNV method eliminates spectral errors caused by sample scattering, optical path variations, and other factors; performs independent standardization correction on each spectrum; and thereby enhances the comparability and stability of data [22]. The purpose of MSC is to eliminate scattering effects, enhance the spectral information related to chemical components, and improve model prediction accuracy through linear regression [23]. Application of the three spectral preprocessing methods furnished a dependable data groundwork for the ensuing model building.

#### 2.4.2. Dataset Partitioning

In this study, the sample set partitioning based on joint x-y distances (SPXY) algorithm was employed to divide all samples into calibration and prediction sets at a ratio of 3:1 according to the joint x-y distance. The calibration set was used for model construction and internal cross-validation, while the prediction set was used for external validation to evaluate the generalization performance of the model [24].

To avoid cultivar information leakage, the SPXY iterative partitioning was performed using “cultivar” as the selection unit: after calculating the joint distances of the 180 spectral samples, cultivars were selected one by one, and once a cultivar was selected, all three of its corresponding spectra were assigned together to either the calibration set or the prediction set. This ensures that the calibration and prediction sets are mutually exclusive at the cultivar level, thereby enabling a more realistic assessment of the model’s predictive ability for “unknown cultivars”.

### 2.5. Feature-Variable Extraction

Spectral data are characterized by high dimensionality and large volume. By selecting feature variables to construct feature subsets, dimensionality can be effectively reduced and redundant or noisy information eliminated, thereby simplifying model structure and improving prediction accuracy and computational efficiency, while also enhancing model robustness and interpretability. In this study, competitive Adaptive Reweighted Sampling (CARS) algorithm was used to feature-variable extraction, this method simulates the principle of “survival of the fittest” by sequentially employing adaptive reweighting, cross-validation screening, and Monte Carlo iterations to optimally select feature wavelengths, the Monte Carlo sampling was repeated 100 times [25,26].

### 2.6. Prediction Models and Evaluation Indexes

#### 2.6.1. Prediction Models

Partial Least Squares Regression (PLSR), Support Vector Machine (SVM), and Back Propagation Neural Network (BPNN) were employed to construct the prediction models of grape samples in this study.

The PLSR algorithm establishes a linear regression model by extracting latent variables shared by predictors and responses, which is especially suited for spectral data with numerous predictors and high collinearity, allowing quantitative prediction of component contents. In this study, a 5-fold cross-validation was used to determine the optimal number of potential variables. The SVM algorithm, grounded in structural risk minimization, maps data into high-dimensional space via kernel functions, making it suitable for linear and nonlinear modeling and offering strong generalization in both classification and regression. In this study, the radial basis function (RBF) was adopted as the kernel function. A comprehensive hyperparameter search was conducted using Bayesian optimization, with candidate values for the regularization parameter C set to [0.1, 1, 10, 100, 1000] and for the kernel coefficient γ set to [0.0001, 0.001, 0.01, 0.1, 1, 10]. The optimization objective was to maximize the average R_P_^2^ across all cross-validation folds. The BPNN algorithm trains multilayer networks via error backpropagation to achieve complex nonlinear mapping without predefining function forms. With strong adaptability, it is widely used in classification, regression, and pattern recognition, and is among the most common feedforward neural network algorithms, offering excellent fitting performance [27,28,29]. In this study, the network architecture was set to a single hidden layer, with an initial learning rate of 0.01. The Sigmoid function was selected as the activation function, and the Levenberg–Marquardt (LM) algorithm was adopted as the training algorithm. Additionally, we applied early stopping with a patience of 20 epochs, monitoring the validation loss with a minimum delta of 0.001.

#### 2.6.2. Evaluation Indexes

Model performance was evaluated using the coefficient of determination (*R*^2^), root mean squared error (*RMSE*), and residual predictive deviation (*RPD*), with calculations performed according to Equations (6)–(8).

(6) $$R^{2}=1-\frac{\sum_{i=1}^{n}{\left(X_{Pi}-X_{i}\right)}^{2}}{\sum_{i=1}^{n}{\left(X_{i}-\bar{X}\right)}^{2}}$$

(7) $$RMSE=\sqrt{\frac{\sum_{i=1}^{n}{\left(X_{Pi}-X_{i}\right)}^{2}}{n}}$$

(8) $$RPD=\frac{1}{\sqrt{1-R^{2}}}$$

An *R*^2^ below 0.6 indicated poor reliability, while values between 0.6 and 0.8 denoted good predictive ability, and values exceeding 0.8 reflected excellent performance. Lower *RMSE* values corresponded to higher prediction accuracy. For *RPD*, values below 1.5 suggested insufficient predictive power, values between 1.5 and 2.0 indicated moderate quantitative capability, and values greater than 2.0 signified outstanding predictive performance and practical applicability [30,31].

## 3. Results

### 3.1. Statistics of Chemical Measurement Values and Dataset Partitioning

#### 3.1.1. Statistics of Chemical Measurement Values

**Figure 1:**
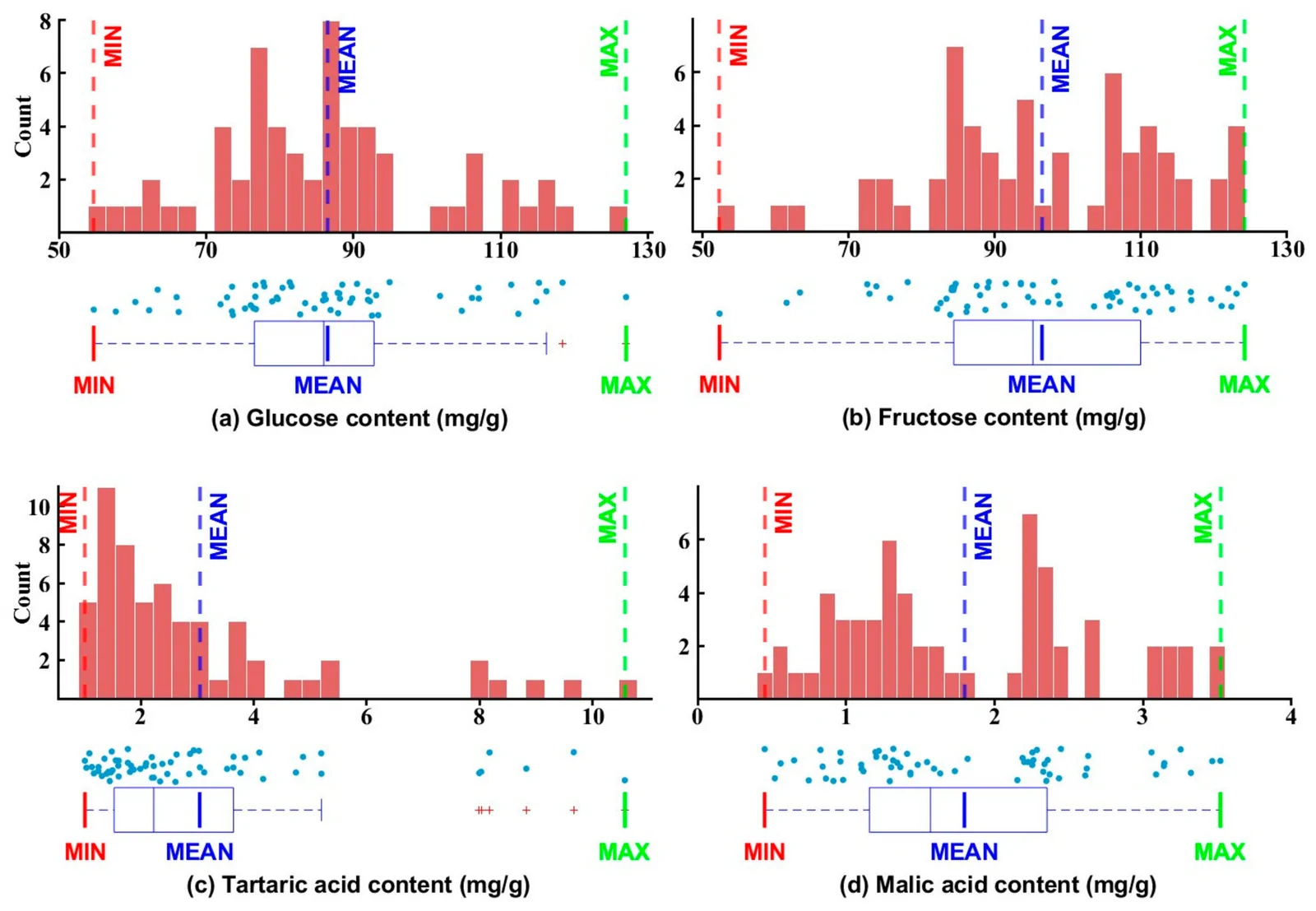
Frequency distribution histogram and scatter plot of grape sample components. (**a**) Glucose, (**b**) fructose, (**c**) tartaric acid, (**d**) malic acid.

In this experiment, the distribution of glucose, fructose, tartaric acid, and malic acid contents in samples of 60 mature grape varieties were determined, and the results are shown in Figure 1.

Among the 60 samples, the glucose content ranged from 54.74 to 127.00 mg/g, with a mean of 86.49 mg/g, a standard deviation (SD) of 15.94 mg/g, and a coefficient of variation (CV) of 18.43%. The fructose content ranged from 52.31 to 124.18 mg/g, with a mean of 96.45 mg/g, a SD of 16.70 mg/g, and a CV of 17.31%. The tartaric acid content ranged from 1.01 to 10.57 mg/g, with a mean of 3.04 mg/g, an SD of 2.25 mg/g, and a CV of 74.10%. The malic acid content ranged from 0.45 to 3.52 mg/g, with a mean of 1.80 mg/g, a SD of 0.83 mg/g, and a CV of 46.20%.

Based on the content distribution, the sugar-to-acid ratio of this sample group exceeded 36.0, generally meeting the characteristics of table grapes or high-quality wine-making materials. The mean fructose content was higher than that of glucose, indicating that the berries had reached full maturity. The tartaric acid to malic acid ratio was approximately 1.65, which is consistent with the typical organic acid profile of *Vitis vinifera* grapes, characterized by a predominance of tartaric acid [32]. Furthermore, the substantial variability in component contents among samples provides rich gradient information, offering a favorable foundation for subsequent model construction and optimization of generalization performance.

#### 3.1.2. Statistics of Dataset Partitioning Results

In this study, 180 samples were divided into calibration set and prediction set at a ratio of 3:1 using SPXY method. The results were shown in Table 1, where the calibration set comprised 135 samples and the prediction set included 45 samples.

The reference measurement ranges for glucose in the calibration and prediction sets were 56.11–119.02 mg/g and 70.33–113.30 mg/g; for fructose in the calibration and prediction sets were 71.18–116.26 mg/g and 61.49–123.08 mg/g; for tartaric acid in the calibration and prediction sets were 0.56–7.08 mg/g and 1.01–8.03 mg/g; and for malic acid in the calibration and prediction sets were 0.80–3.06 mg/g and 0.45–3.47, respectively. In the prediction set and calibration set, the means and standard deviations of all measured parameters were comparable, indicating a balanced data distribution and the absence of significant sample selection bias, thereby satisfying the basic requirement of distributional consistency for subsequent modeling.

### 3.2. Spectral Characterization of Grape Samples

**Figure 2:**
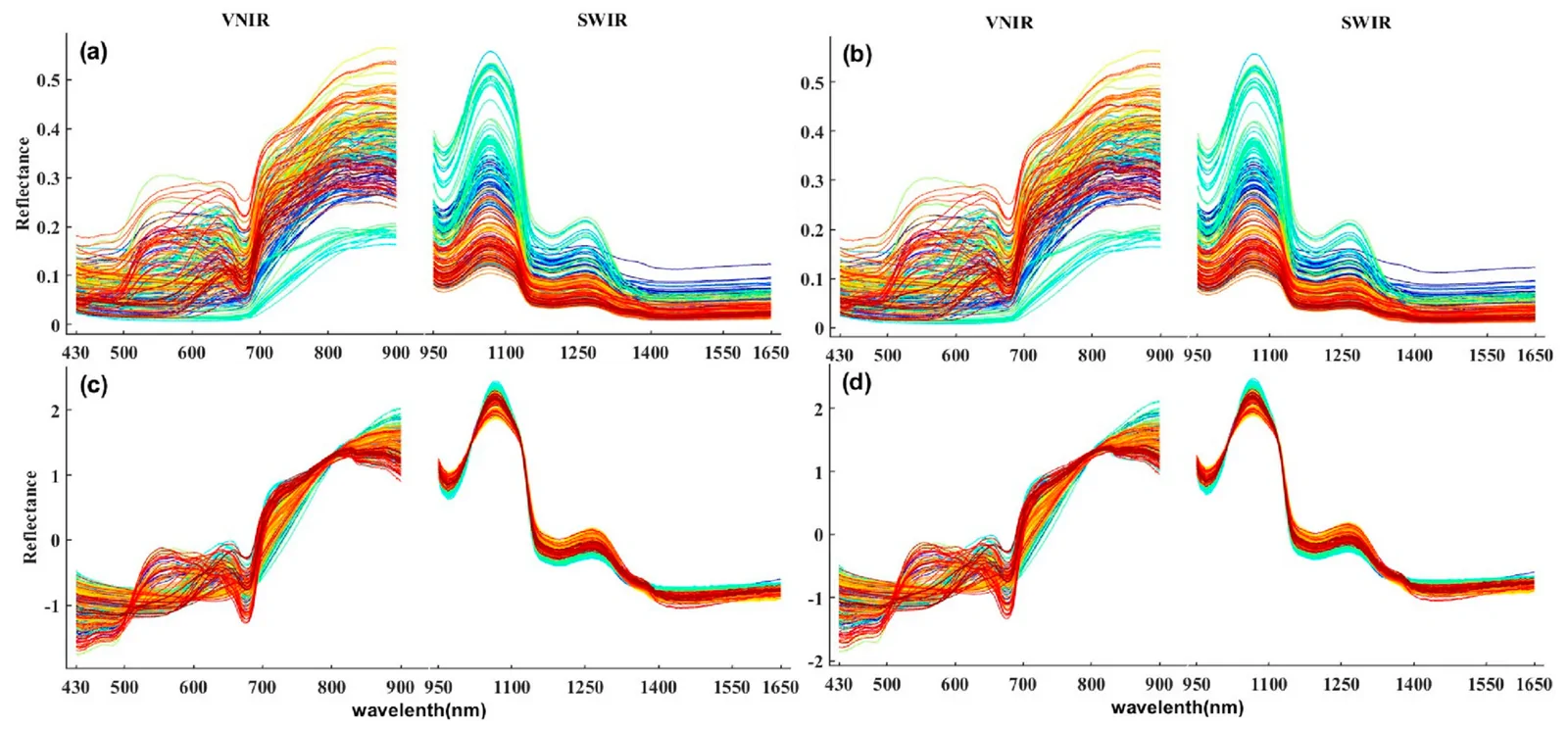
Spectral curves of grape samples. (**a**) Raw, (**b**) S-G, (**c**) S-G-SNV, (**d**) S-G-SNV-MSC.

Figure 2 illustrates the raw and preprocessed spectral curves of 180 grape samples. Figure 2a presents the original spectral reflectance curves of the samples. The spectral profiles in the VNIR and SWIR bands were generally consistent, conforming to the typical spectral response characteristics of grape berries [33]. However, the significant differences in reflectance among varieties are mainly attributable to differences in their skin color and internal chemical composition. For example, in the VIS region, the spectral curve exhibits a reflectance peak at around 530 nm and an absorption valley at around 670 nm. This “green-peak, red-valley” feature is closely related to the optical response of pigments (e.g., anthocyanins and chlorophylls) present in grape skins [34]. In the NIR and SWIR regions, the formation of spectral peaks and valleys may be related to the stretching vibrations of C-H and O-H bonds [35].

The raw spectral curves were sequentially preprocessed using S-G, SNV, and MSC algorithms, and the results are shown in Figure 2b–d; the effectiveness of this combined preprocessing strategy has been validated in multiple studies [21,22,23]. Through a systematic preprocessing procedure involving smoothing followed by scatter correction, the signal-to-noise ratio and consistency of the spectral data were effectively improved, thereby laying a solid foundation for the accuracy and robustness of subsequent modeling. Taking the PLSR modeling of glucose as an example, Table 2 compares the effects of these three preprocessing strategies.

Compared with the full-spectrum model, all preprocessing schemes improved the predictive performance, among which the combined application of S-G, SNV, and MSC achieved the best results. This conclusion also holds true for the PLSR models of fructose, tartaric acid, and malic acid, which demonstrates the effectiveness of the combined preprocessing strategy. Therefore, the combined strategy of S-G, SNV, and MSC was adopted for all subsequent preprocessing methods.

### 3.3. Feature Variables Analysis by CARS Algorithm

**Figure 3:**
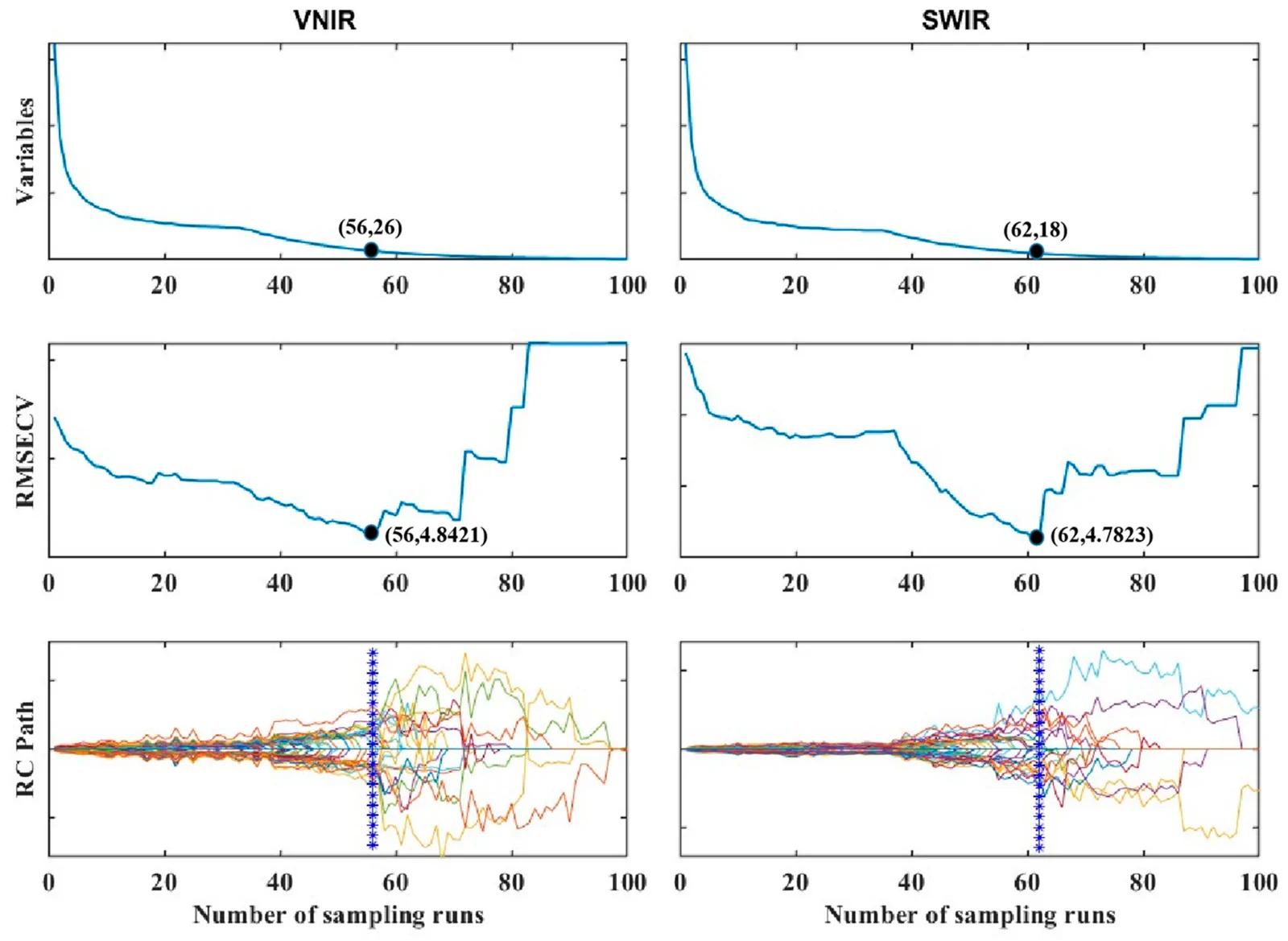
Process of extracting glucose feature variables based on the CARS algorithm.

The CARS algorithm was employed to extract feature wavelengths closely related to glucose, fructose, tartaric acid, and malic acid within the VNIR and SWIR spectral regions, respectively, and to determine the optimal variable subset for each component based on the minimum RMSE_CV_ criterion. As the number of Monte Carlo sampling iterations increased, the number of extracted variables first decreased exponentially and then gradually stabilized, and the RMSE_CV_ values exhibited a trend of first decreasing and then increasing. The initial decrease indicates that irrelevant variables were effectively eliminated, while the subsequent increase suggests that irrelevant variables were reintroduced, which interfered with predictive performance. Therefore, the feature-variable subset corresponding to the minimum RMSE_CV_ value is considered the optimal subset. The feature subsets selected from the VNIR and SWIR bands were fused to serve as the optimal variable set for constructing the prediction model. Figure 3 illustrates the iteration process of glucose feature variables.

In the VNIR range, when the number of Monte Carlo iterations reached 56, the RMSECV decreased to its minimum value of 4.8421, with 26 feature variables selected, accounting for 4.02% of the total 646 variables. In the SWIR range, the RMSE_CV_ reached its lowest value of 4.7823 at 62 iterations, with 18 variables extracted, representing 12.16% of the total 148 variables. By repeating the above extraction procedure, the feature-variable selection results for fructose, tartaric acid, and malic acid were obtained, as shown in Table 3.

Ultimately, the optimal feature-variable subsets for glucose, fructose, tartaric acid, and malic acid contained 44, 31, 35, and 39 variables, respectively. Subsequently, PLSR models were constructed using all variables and the optimal subset, respectively, and their performance was compared, as shown in Table 4.

Compared with the whole-variable-PLSR model, the CARS-PLSR model performed better on all four indices, namely glucose, fructose, tartaric acid, and malic acid: the R_P_^2^ increased by 0.0431, 0.0427, 0.0266, and 0.0698, respectively; the RMSE_P_ decreased by 0.3407, 0.3547, 0.045, and 0.0497, respectively; and the RPD_P_ increased by 0.1138, 0.0993, 0.0574, and 0.1705, respectively. The above results demonstrate that the CARS algorithm can significantly reduce the feature-variable while preserving key information, thereby lowering model complexity and improving prediction accuracy.

### 3.4. Comparison of Prediction Models and Optimal Prediction Models

#### 3.4.1. Comparison of Prediction Models

**Figure 4:**
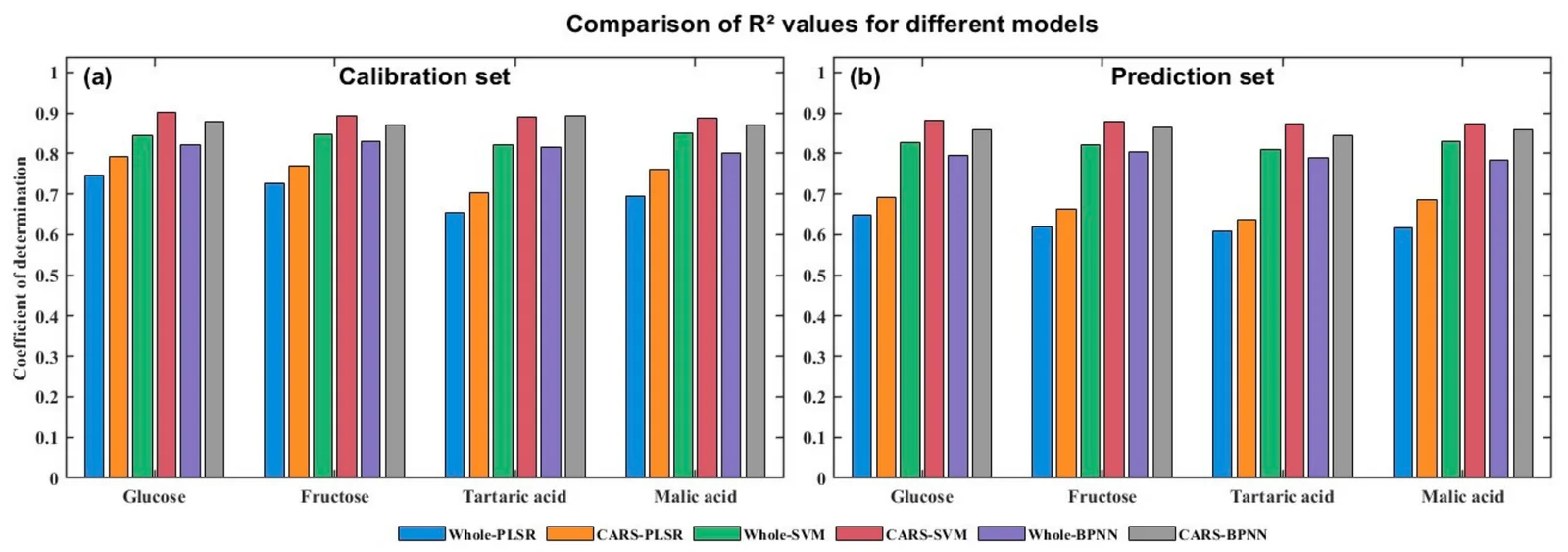
Comparison of R^2^ for different models. (**a**) R_C_^2^; (**b**) R_P_^2^.

**Figure 5:**
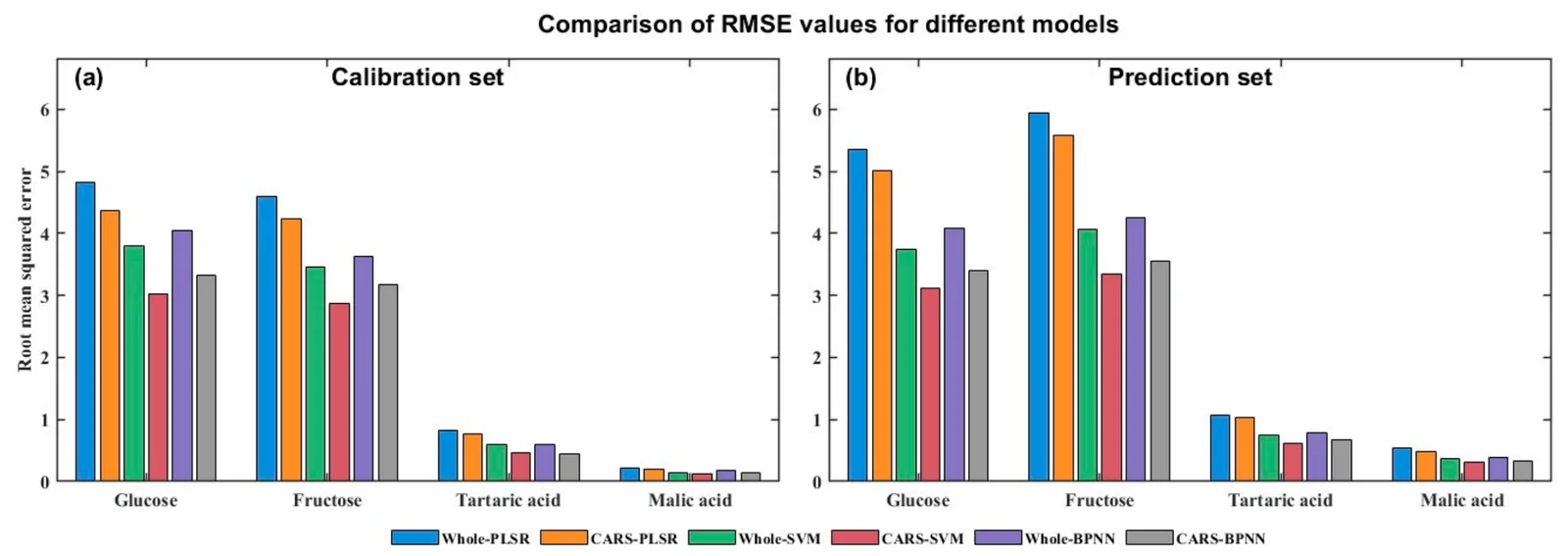
Comparison of RMSE for different models. (**a**) RMSE_C_; (**b**) RMSE_P_.

**Figure 6:**
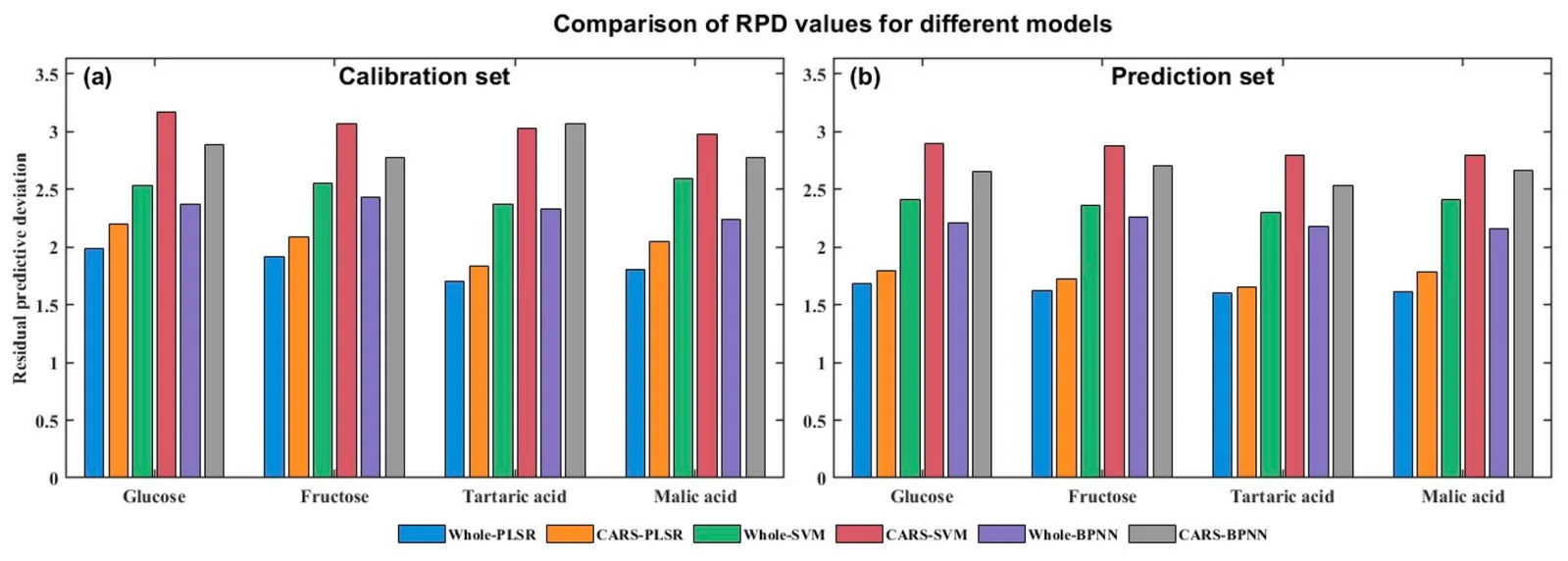
Comparison of RPD for different models. (**a**) RPD_C_; (**b**) RPD_P_.

In this study, PLSR, SVM, and BPNN prediction models were established for all four indices using whole-variable and feature-variable subsets, respectively. The prediction results of all models are presented in Figure 4, Figure 5 and Figure 6.

Figure 4 compares the R^2^ of six models for glucose, fructose, tartaric acid, and malic acid. The results show that, for the same modeling algorithm, the CARS feature-variable models all achieved higher prediction accuracy than the whole-variable models, indicating that feature-variable selection is effective. Among different algorithms, the nonlinear models SVM and BPNN both outperformed the linear PLSR model. Among them, the CARS-SVM model exhibited the best prediction performance for glucose, fructose, tartaric acid, and malic acid, with R_P_^2^ of 0.9002, 0.8939, 0.8909, and 0.8873, respectively.

Figure 5 compares the RMSE of six models for the four indices. Under the same algorithm, the RMSE values of the CARS feature-variable models were all lower than those of the whole-variable models, indicating that feature selection effectively reduced prediction errors. Among different models, the RMSE values of SVM and BPNN were both lower than that of PLSR, and the CARS-SVM model achieved the lowest RMSE for all four indices, demonstrating the best performance. The absolute RMSE values for glucose and fructose were relatively large, while those for tartaric acid and malic acid were smaller, which is related to differences in component concentration levels; however, the performance trends among models were consistent across indices.

Figure 6 shows the comparison of RPD values among six models for four indices. Under the same algorithm, the RPD values of the CARS feature-variable models were all higher than those of the whole-variable models, indicating that feature selection effectively improved prediction ability. Among different algorithms, the RPD values of SVM and BPNN were generally superior to those of PLSR. Notably, the CARS-SVM model achieved RPD values exceeding 2.7 for all four indices, demonstrating the best performance.

Furthermore, from an overall trend perspective, the calibration set performed slightly better than the prediction set, indicating that the model did not exhibit obvious overfitting and possessed good generalization ability [36].

#### 3.4.2. The Optimal Prediction Models

**Figure 7:**
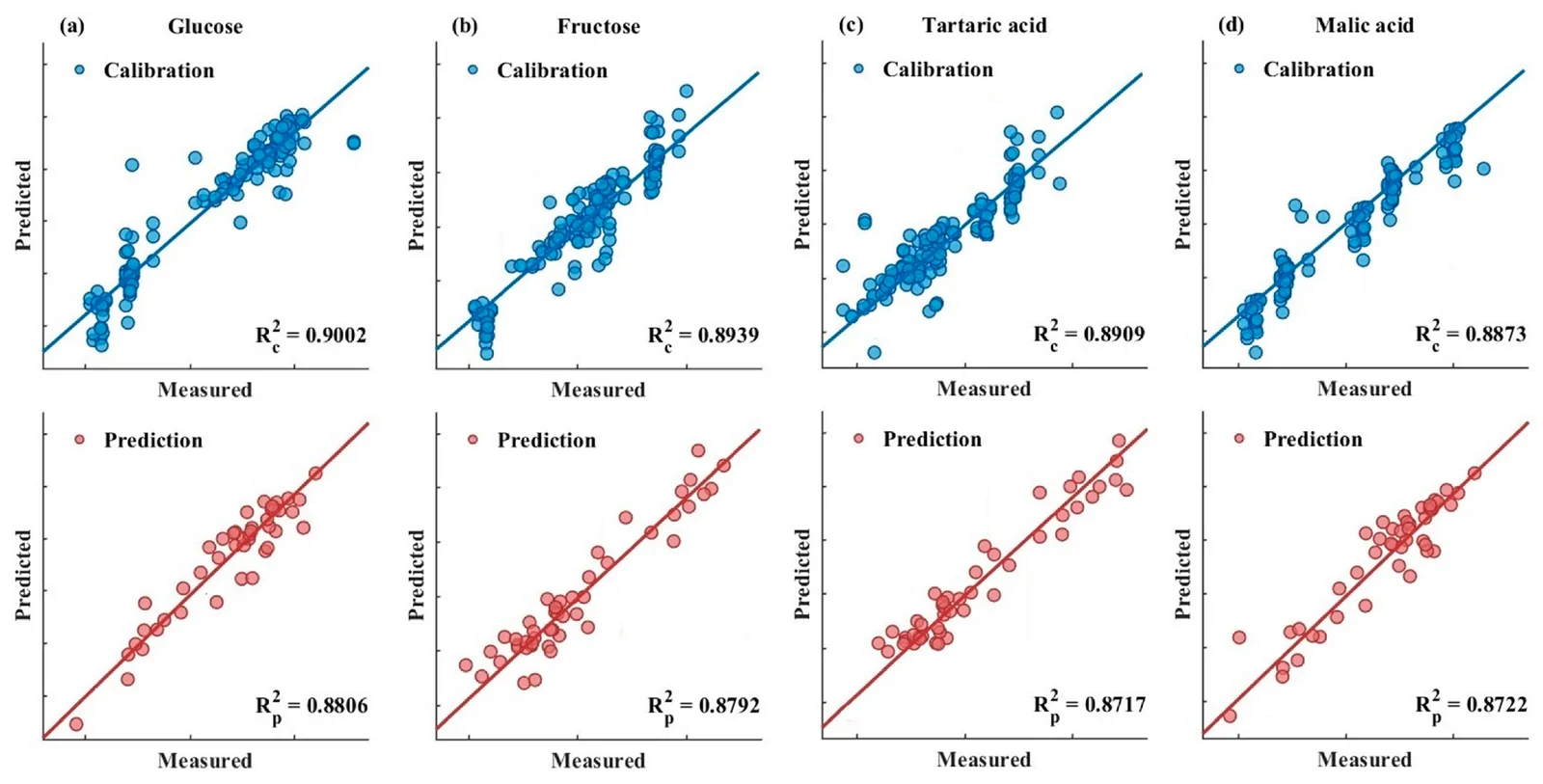
Fitting results of CARS-SVM for calibration set and prediction set. (**a**) Glucose, (**b**) fructose, (**c**) tartaric acid, (**d**) malic acid.

In this study, PLSR, SVM, and BPNN prediction models were established for four grape components based on full variables and feature variables, respectively. The results showed that the feature-variable models outperformed the full-variable models in prediction performance, confirming the effectiveness of feature subset selection. Among them, the linear CARS-PLSR models all had R_P_^2^ values greater than 0.7 and RPD_P_ values exceeding 1.65, indicating a moderate level of quantitative analysis capability. In contrast, the nonlinear CARS-SVM and CARS-BPNN models all had R_P_^2^ values approaching or exceeding 0.8 and RPD_P_ values exceeding 2.5, demonstrating excellent quantitative analysis capability. This can be attributed to the ability of nonlinear models to better fit the complex relationship between grape spectra and component concentrations through kernel mapping or nonlinear networks. Moreover, the CARS-SVM model achieved slightly better prediction performance than the CARS-BPNN model. Figure 7 shows the fitting results of the optimal CARS-SVM model for glucose, fructose, tartaric acid, and malic acid.

As can be seen from the figure, the scatter points are closely distributed on both sides of the fitting lines for the calibration set and prediction set, indicating that the model did not exhibit overfitting and possesses good predictive ability and robustness. Ultimately, the CARS-SVM model performed best, with R_P_^2^ values for glucose, fructose, tartaric acid, and malic acid reaching 0.9002, 0.8939, 0.8909, and 0.8873; RPD_P_ values reaching 2.8940, 2.8772, 2.7918, and 2.7973; and RMSE_P_ values as low as 3.1186, 3.3419, 0.6145, and 0.3051, respectively.

It is noteworthy that for fructose and tartaric acid, the concentration distribution in the calibration set is slightly uneven, with fewer samples at the high-concentration end. This may result in minor extrapolation errors when predicting the highest concentration samples in the prediction set. Nevertheless, the overall prediction accuracy remains highly satisfactory for practical applications.

## 4. Discussion

### 4.1. Discussion on Grape Samples

#### 4.1.1. Influence of Sample Representativeness and Diversity on Model Generalization

A total of 60 grape varieties were selected as the research objects in this study, covering two major categories: table grapes and wine grapes. The skin colors involved multiple color series, including red, pink, purplish red, yellowish green, and blue-black. This sampling scheme exhibits considerable diversity in genetic background, cultivation purpose, and appearance traits, which can reduce model bias caused by a single sample type, provide a reliable data foundation for the construction and validation of quantitative models for grape spectral components, and help improve the generalization ability and universality of the established models [37].

#### 4.1.2. Constraints of Chemical Reference Value Accuracy on Quantitative Modeling

In this study, chemical reference values were used as the reference for spectral quantitative modeling. The contents of glucose, fructose, tartaric acid, and malic acid in all grape samples were determined by HPLC, with repeated measurements averaged for each sample to ensure the accuracy and reproducibility of the reference values [19]. The contents of the four components obtained from chemical determination served as the target variables for subsequent modeling and corresponded to the spectral data of the samples. The accurate determination of component contents directly determines the upper limit of model performance and serves as the foundation for the reliability of spectral quantitative modeling [38].

Note that the HPLC reference values were obtained from 15 berries, whereas the spectral scans were acquired from a different set of 15 berries from the same cluster. This indirect matching of spectra to reference values may introduce sampling noise due to berry-to-berry biological variability within a cluster. Such intra-cluster variance acts as an additional random error component in the reference values and can therefore inflate the reported RMSE. Even if the spectral model were unbiased, the mismatch between the scanned berries and those used for HPLC reference determination would contribute to prediction error, leading to a conservative—i.e., upward-biased—RMSE estimate. In addition, random errors in the reference values can attenuate the regression slope and reduce R^2^. Because the same reference-matching protocol was used for all models, the relative comparison among CARS-PLSR, CARS-SVM, and CARS-BPNN remains valid, although the absolute RMSE values should be interpreted with caution.

The intra-cluster variance reflects biological and sampling variability among berries within the same cluster and is treated as noise in the response variable y. Consequently, the reported RMSE includes this biological sampling error and should not be regarded solely as instrumental or model prediction error. Future studies should ideally perform spectral acquisition and HPLC analysis on the same individual berries or use a larger number of homogenized berries per cluster to better represent the cluster average.

### 4.2. Discussion on Feature Variables Extracted by CARS

**Figure 8:**
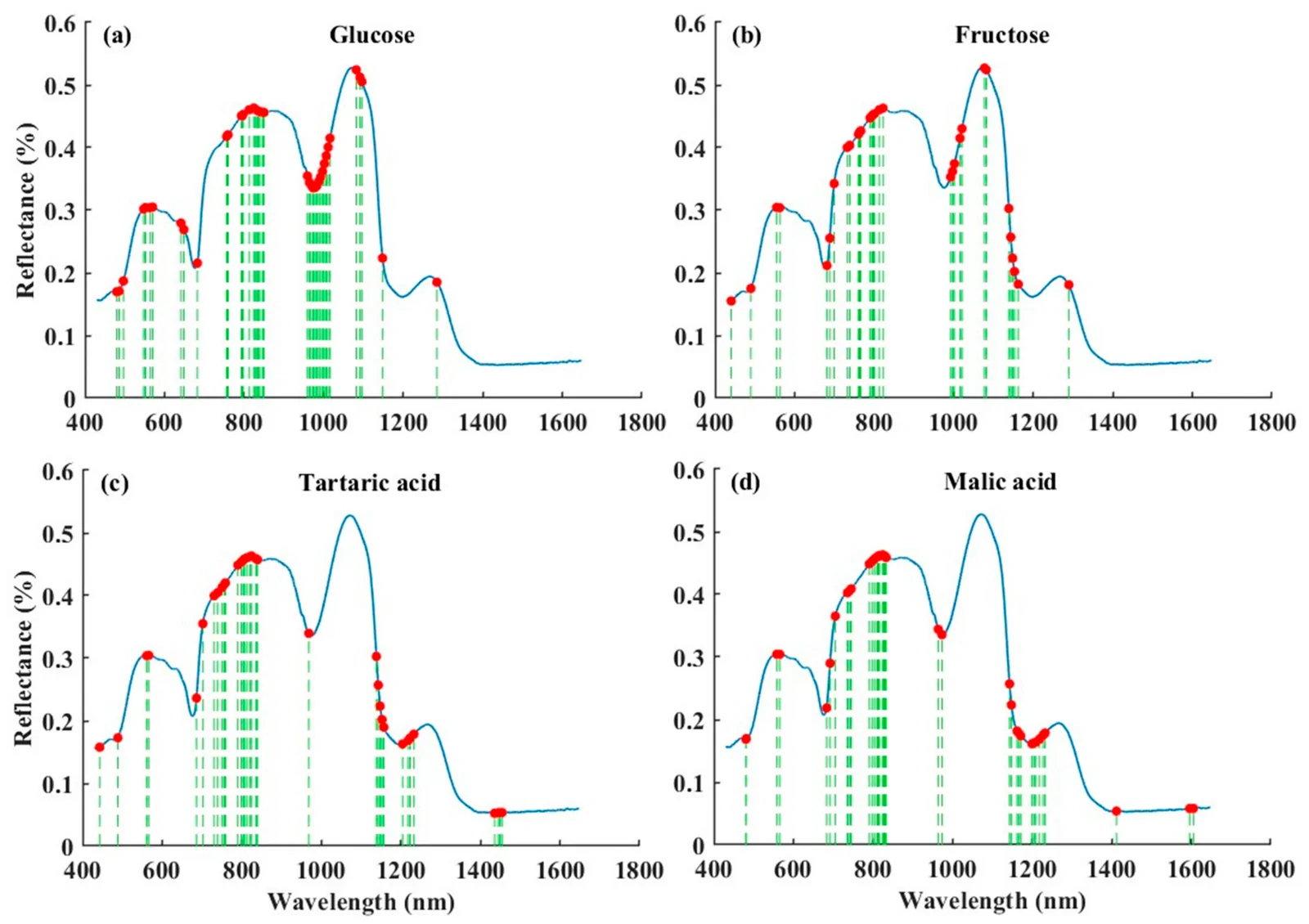
Distribution of feature variables selected by CARS. (**a**) Glucose, (**b**) fructose, (**c**) tartaric acid, (**d**) malic acid.

In this study, the CARS feature extraction method was employed, with the minimum RMSECV as the criterion, to effectively reduce the data dimensionality. CARS feature selection significantly reduced the data dimensionality for each component. The number of feature variables for glucose, fructose, tartaric acid, and malic acid was compressed from 794 to 44, 31, 35, and 39, respectively, with reductions all exceeding 94%, among which fructose showed the largest decrease of 96.10%. Models built with the selected feature variables all outperformed the full-variable models, indicating that the variables screened by CARS have the highest correlation with each component and can effectively eliminate redundant information and interference, thereby simplifying the models and enhancing their robustness and generalization ability [39]. Figure 8 shows the distribution of feature variables for glucose, fructose, tartaric acid, and malic acid.

From the distribution ranges of the selected feature variables, the feature variables for glucose and fructose were mainly concentrated in three regions: 720–840 nm, 950–1020 nm, and 1080–1150 nm. Among them, 720–840 nm was mainly attributed to C-H stretching vibrations, 950–1020 nm mainly corresponded to the second overtone of O-H stretching vibrations, and 1080–1150 nm mainly corresponded to the second overtone of C-H stretching vibrations. Tartaric acid and malic acid feature variables were mainly distributed at 780–840, 1150–1230, and around 1430 nm, with relatively weaker signals than sugars, yet all related to C-H and O-H stretching vibrations. In summary, the CARS feature extraction method combines efficiency and superiority, simultaneously improving prediction performance and simplifying model structure.

In addition, a small number of feature variables were distributed in the visible region of 430–700 nm. Sugars and organic acids do not exhibit electronic transitions in this wavelength range; therefore, the selection of these wavelengths may be attributed to the indirect transmission of sugar and acid information through differences in variety and color [40,41]. When screening variables based on statistical correlation, the CARS algorithm retained some visible wavelengths that were sufficiently statistically correlated with sugar and acid contents [42].

To eliminate this risk of spurious association, we re-executed the complete modeling workflow while strictly excluding the visible region (430–700 nm). CARS was rerun on the spectral matrix containing only NIR/SWIR variables, and the CARS-PLSR, CARS-SVM, and CARS-BPNN models were retrained and evaluated using the same calibration/prediction-set split, preprocessing methods, and hyperparameter optimization strategy as in the original analysis. Taking glucose prediction as an example, the results showed that the CARS-PLSR, CARS-SVM, and CARS-BPNN models using only NIR/SWIR variables achieved R_P_^2^ values of 0.6892, 0.8630, and 0.8206; RMSE_P_ values of 5.3402, 3.5114, and 4.0630; and RPD_P_ values of 1.7937, 2.7287, and 2.3611, respectively. Compared with the full-spectrum models, the prediction performance of the above models decreased only slightly but still maintained high prediction accuracy; among them, the NIR/SWIR-CARS-SVM model performed best, and the other components showed the same trend. Out of caution, we included only NIR/SWIR variables in the final model specification, adopting a more conservative variable-selection strategy that eliminated the risk of spurious associations and made the interpretation of the results more robust.

### 4.3. Discussion on Prediction Model Performance

Regarding predictive accuracy, the nonlinear SVM and BPNN approaches surpassed the linear PLSR model. This discrepancy reflects differences in how these algorithms accommodate data structure and complexity, particularly the restricted capacity of linear PLSR to capture nonlinear feature–response relationships [43,44,45]. Nevertheless, the relationship between grape spectral response and component concentration is inherently nonlinear, largely because grape berries are high-moisture, multi-constituent biological matrices. The spectral response depends not only on the concentrations of glucose, fructose, tartaric acid, and malic acid, but is also influenced by multiple factors such as strong water absorption, intermolecular hydrogen bonding between sugars and acids, component interactions, light scattering, physical properties of the berry surface, and baseline drift, causing the spectra to deviate from a strictly linear relationship with chemical values. In addition, severe peak overlap and high collinearity among variables in the near-infrared and shortwave near-infrared regions further exacerbate the nonlinearity and interaction effects of the spectral response. Therefore, linear PLSR models struggle to fully characterize this complex mapping relationship, whereas nonlinear SVM and BPNN models can achieve high-dimensional nonlinear approximation through kernel functions or hidden layers, thereby extracting valid information from the spectra more effectively. After feature-variable selection by CARS, model dimensionality is reduced and noise and redundancy are diminished, further highlighting the advantages of nonlinear models.

Regarding the two nonlinear models, the SVM model showed slightly better prediction performance than the BPNN model. This difference may be attributable to the following factors. First, the sample size in this study was relatively limited. SVM, following the structural risk minimization principle, exhibits good generalization capability under small-sample conditions, whereas BPNN, with its numerous parameters, generally requires a large sample for adequate training. Second, SVM obtains a global optimum by solving a convex quadratic programming problem, yielding stable and reproducible results; BPNN relies on gradient descent and is sensitive to initial weights, learning rate, and network architecture, making it prone to converging to local minima and causing noticeable performance fluctuation. Third, after CARS selects a small number of key wavelengths, data dimensionality and redundancy are reduced. SVM can fully exploit these features and remain stable with limited samples, while BPNN may suffer from insufficient information or parameter redundancy, leading to degraded generalization capability. In addition, SVM exhibits stronger robustness and a certain tolerance to outliers and noise [46,47]. Therefore, under the conditions of this study—such as limited sample size, high-dimensional collinearity of spectra, and CARS feature selection—SVM outperforms BPNN in both prediction accuracy and stability, owing to its strong small-sample generalization, global optimality, and robustness [48].

### 4.4. Limitations and Scope of Inference

This study was conducted at a single location (Taigu Grape Nursery, Shanxi) during one harvest season. Although the 60-variety panel provides substantial genetic breadth, the absence of multi-environment and multi-year data means that genotype, environment interactions and interannual climate variability could not be assessed. The findings should therefore be regarded as a first-step characterization under the specific conditions of this trial, and conclusions regarding universal industrial applicability are not intended. Future work will extend this design to multiple sites and vintages to test the stability and transferability of the observed variety-level patterns.

## 5. Conclusions

This study demonstrates that combining VNIR and SWIR spectroscopy with chemometrics enables accurate and non-destructive quantitative analysis of sugar and acid components in grapes. Based on spectral preprocessing and feature-variable selection, the PLSR, SVM, and BPNN prediction models constructed for sugar and acid components all outperformed the whole-variable models. Among them, the NIR/SWIR-CARS-SVM model performed best, with R_P_^2^ values reaching 0.8630, 0.8441, 0.8546, and 0.8283; RPD_P_ values reaching 2.7287, 2.5782, 2.6451, and 2.4145; and RMSE_P_ values as low as 3.5114, 3.4751, 0.5310, and 0.1646 for glucose, fructose, tartaric acid, and malic acid, respectively.

Overall, this study conducted only a preliminary characterization analysis of 60 grape varieties at a single site and in a single year and has not yet carried out experiments on grape composition across different geographical environments, years, or climatic conditions. Therefore, the variety-level patterns observed should be regarded as generative hypotheses rather than universal conclusions. Future research should include more grape varieties from different production regions, years, and maturity stages to improve the model’s generalization ability and universality, thereby better meeting the needs of industrial application.

## Figures and Tables

**Table 1:** Dataset partitioning and composition of grape samples.

Parameter	Calibration Set					Prediction Set				
	Mean	Max	Min	SD	CV	Mean	Max	Min	SD	CV
Glucose (mg/g)	86.963	119.02	56.114	9.5808	0.1101	87.522	113.30	70.334	9.0344	0.1032
Fructose (mg/g)	97.439	116.26	71.180	8.8144	0.0905	93.482	123.08	61.491	9.6102	0.1028
Tartaric acid (mg/g)	3.1472	7.0846	0.5565	1.3917	0.4422	2.7292	8.0274	1.0103	1.7123	0.6274
Malic acid (mg/g)	1.8070	3.0557	0.8012	0.3973	0.2199	1.7663	3.4660	0.4514	0.8586	0.4861

**Table 2:** Results of the PLSR model for glucose content by spectral pretreatment method.

Parameter	Preprocessing	Calibration Set			Prediction Set		
		R_C_^2^	RMSE_C_	RPD_C_	R_P_^2^	RMSE_P_	RPD_P_
Glucose	Raw	0.6967	5.2817	1.8158	0.5875	5.8012	1.5570
	S-G	0.7076	5.1835	1.8493	0.6020	5.6991	1.5851
	S-G + SNV	0.7279	5.0029	1.9171	0.6269	5.5185	1.6371
	S-G + SNV + MSC	0.7473	4.8858	1.9893	0.6482	5.3623	1.6860

**Table 3:** Feature variables proposed by CARS algorithm.

Parameter	VNIR (646 Variables)			SWIR (148 Variables)		
	Iterations	Number of Variables	RMSE_CV_	Iterations	Number of Variables	RMSE_CV_
Glucose	56	26	4.8421	62	18	4.7823
Fructose	64	18	4.5225	75	13	4.6286
Tartaric acid	33	21	0.7951	52	14	0.8048
Malic acid	51	23	0.3515	46	16	0.3901

**Table 4:** Prediction results of PLSR model based on whole-variable and CARS-selected subset.

Parameter	Method	Number of Variables	Calibration Set			Prediction Set		
			R_C_^2^	RMSE_C_	RPD_C_	R_p_^2^	RMSE_p_	RPD_p_
Glucose	Whole	794	0.7473	4.8858	1.9893	0.6482	5.3623	1.6860
	CARS	44	0.7927	4.3614	2.1963	0.6913	5.0216	1.7998
Fructose	Whole	794	0.7271	4.6004	1.9142	0.6192	5.9336	1.6205
	CARS	31	0.7702	4.2289	2.0861	0.6619	5.5789	1.7198
Tartaric acid	Whole	794	0.6544	0.8198	1.7010	0.6096	1.0711	1.6005
	CARS	35	0.7031	0.7623	1.8352	0.6362	1.0261	1.6579
Malic acid	Whole	794	0.6947	0.2236	1.8098	0.6163	0.5312	1.6144
	CARS	39	0.7614	0.2032	2.0472	0.6861	0.4815	1.7849

## Data Availability

The original contributions presented in this study are included in the article. Further inquiries can be directed to the corresponding authors.

## Abbreviations

The following abbreviations are used in this manuscript:

VNIR	Visible and Near-Infrared
SWIR	Short-Wave Infrared
PLSR	Partial Least Squares Regression
SVM	Support Vector Machine
BPNN	Back Propagation Neural Network
CARS	Competitive Adaptive Reweighted Sampling
HPLC	High-performance Liquid Chromatography
